# Cancer Risk Assessment: Chemical Carcinogenesis, Hazard Evaluation, and Risk Quantification

**DOI:** 10.1289/ehp.119-a312

**Published:** 2011-07-01

**Authors:** Raymond D. Harbison

**Affiliations:** Raymond Harbison has > 30 years of experience as a toxicologist, pharmacologist, and human health risk assessor. He is currently Professor of Environmental and Occupational Health, at the College of Public Health, and Professor of Pathology and Pharmacology, at the College of Medicine, University of South Florida, Tampa, Florida. He is also Adjunct Professor of Medicine at the State University of New York, College of Medicine at Buffalo, New York. He directs the Center for Environmental/Occupational Risk Analysis and Management for the identification and control of environmental, occupational, and agricultural diseases, and maintains an active laboratory for elucidating the mechanisms of the chemical causes of organ injury and disease.

Humans can be exposed to a panoply of agents and actions including chemicals, nanomaterials, metals, and complex mixtures. The link between these exposures and possible clinical sequelae including cancer is often poorly understood. Historically, regulators have addressed such uncertainty by applying linear low-dose defaults when establishing cancer risk estimates for suspected or known carcinogens. However, in recent years, cancer risk assessments have become increasingly sophisticated. An important component of this increased sophistication is the relatively new ability to consider the mode of action by which a substance might exert procarcinogenic, anticarcinogenic, or neutral effects. Knowledge regarding the mode of action can elucidate the relevance to human health associated with the particular exposure. In addition, technological advances in molecular biology and computational analyses increasingly contribute to screening for the potential for substances to cause cancer, or to inform particular areas of risk assessments. *Cancer Risk Assessment* provides an excellent synthesis of the aforementioned topics, as well as others essential for students becoming acquainted with the field or practitioners struggling to stay abreast of the rapid pace of emerging concepts in the literature.

This carefully edited book represents a multiauthor compilation of chapters contributed by well-known experts in the field. The author roster is well-balanced across all sectors of the scientific community, with primary contributions from academia, consulting companies, and government agencies. The book is divided into six major parts presented in a logical format and amenable to the classroom setting or individual study.

A historical and current overview of cancer risk assessment is provided in Part I. The science and regulatory processes that lead to policy decisions are discussed, along with shaping chemical-control frameworks such as the European Commission’s Registration, Evaluation, Authorisation and Restriction of Chemicals (REACH) regulation.

Part II begins with a historical account of cancer, followed by advances in the cell biology of cancers. The classic and mechanistically groundbreaking studies on polycyclic aromatic hydrocarbons (PAHs) are used to convey critical concepts on multistage carcinogenesis, including initiation, promotion, and progression. From there, specific types of dose–response relationship, such as the sometimes controversial hormesis mechanism and always controversial biological thresholds for genotoxic carcinogens, are discussed, thereby supplementing the understanding of these latter critical concepts.

Part III focuses on specific types of genotoxicity testing. Although methodological and analytical aspects of this topic have been addressed extensively in available books, *Cancer Risk Assessment* provides the finest presentation I have seen on regulatory aspects of genotoxicity, specific genotoxicity assays, and interpretative guidance for the various assays. The wealth of information conveyed on this important topic alone justifies purchasing this book.

In Part IV, the reader is introduced to a specific framework for analyzing mode of action and human relevance of chemical-induced tumors. Specific examples are discussed, including PPAR-〈 and liver tumors, 〈_2u_-globulin nephropathy, chronic progressive nephropathy, and urinary tract calculi and bladder tumors. One notable limitation in this part was the absence of a chapter devoted to thyroid follicular cell tumors. Because the mode of action for these types of tumors has been incorporated into regulatory guidance, Part IV is incomplete without presentation on this topic.

Emerging technologies in cancer risk assessment are elucidated in Part V. The section opens with one of the most concise yet comprehensive discussions available on quantitative structure–activity relationship (QSAR) evaluations. This chapter is a *tour de force* for readers engaged in the research and development of new molecules. From QSARs, the reader is transitioned to physiologically based pharmacokinetic (PBPK) models. The application of these models is discussed and includes inter- and intraspecies extrapolations, route-to-route exposure extrapolations, and highly challenging extrapolations from individual carcinogens to mixtures. Specific case studies are also presented. Thereafter, genomic applications to risk assessment are discussed, along with case studies. Part V concludes with a brief recap on PBPK modeling and discusses future research initiatives in computational toxicology.

Part VI describes general approaches for quantifying cancer risks. Quantification based on both linear low-dose- and nonlinear low-dose-extrapolations is presented. These discussions are well balanced and are recommended reading. Of particular interest in this part is a timely presentation and update on the appropriateness of combining neoplasms for the evaluation of rodent carcinogenesis studies. This information will undoubtedly be of significant value to regulatory risk assessors, who have relied up until now on a 1986 publication by a common author of this chapter. Finally, this part concludes with a step-by-step instructional discussion on exposure reconstruction and cancer risk estimation.

In summary, *Cancer Risk Assessment* is a well-written book that reads more like the story of risk assessment than a traditional textbook as one topic flows seamlessly to the next logical point. This book provides extensive coverage in the field, with the topics covered in some cases being so timely that they are not yet available in other texts. However, even topics that have received extensive coverage in other sources are presented in a manner that warrants review. Novice readers and experts alike will benefit from having this comprehensive text as a learning aid or a reliable reference with a well-worn cover.

